# Altered Functionality of Anti-Bacterial Antibodies in Patients with Chronic Hepatitis C Virus Infection

**DOI:** 10.1371/journal.pone.0064992

**Published:** 2013-06-04

**Authors:** Anne Lamontagne, Ronald E. Long, Mary Ann Comunale, Julie Hafner, Lucy Rodemich-Betesh, Mengjun Wang, Jorge Marrero, Adrian M. Di Bisceglie, Timothy Block, Anand Mehta

**Affiliations:** 1 Drexel University College of Medicine, and Department of Microbiology and Immunology and Drexel Institute for Biotechnology and Virology, Doylestown, Pennsylvania, United States of America; 2 Immunotope Inc., Doylestown, Pennsylvania, United States of America; 3 Division of Gastroenterology, University of Michigan, Ann Arbor, Michigan, United States of America; 4 Department of Internal Medicine and Saint Louis University Liver Center, Saint Louis University School of Medicine, St Louis, Missouri, United States of America; University of North Carolina School of Medicine, United States of America

## Abstract

**Background:**

Using comparative glycoproteomics, we have previously identified a glycoprotein that is altered in both amount and glycosylation as a function of liver cirrhosis. The altered glycoprotein is an agalactosylated (G0) immunoglobulin G molecule (IgG) that recognizes the heterophilic alpha-gal epitope. Since the alpha gal epitope is found on gut enterobacteria, it has been hypothesized that anti-gal antibodies are generated as a result of increased bacterial exposure in patients with liver disease.

**Methods:**

The N-linked glycosylation of anti-gal IgG molecules from patients with fibrosis and cirrhosis was determined and the effector function of anti-bacterial antibodies from over 100 patients examined. In addition, markers of microbial exposure were determined.

**Results:**

Surprisingly, the subset of agalactosylated anti-gal antibodies described here, was impaired in their ability to mediate complement mediated lysis and inhibited the complement-mediated destruction of common gut bacteria. In an analysis of serum from more than 100 patients with liver disease, we have shown that those with increased levels of this modified anti-gal antibody had increased levels of markers of bacterial exposure.

**Conclusions:**

Anti-gal antibodies in patients with liver cirrhosis were reduced in their ability to mediate complement mediated lysis of target cells. As bacterial infection is a major complication in patients with cirrhosis and bacterial products such as LPS are thought to play a major role in the development and progression of liver fibrosis, this finding has many clinical implications in the etiology, prognosis and treatment of liver disease.

## Introduction

Worldwide, more than 500 million people have been infected chronically with hepatitis B (HBV) or hepatitis C virus (HCV) [1]. Chronic infection with these viruses leads to liver damage, initially in the form of liver fibrosis [2]. Without intervention, liver fibrosis can progress to cirrhosis and eventually to liver cancer [3].

Although there is a clear association between viral infection and excessive alcohol consumption with the onset of liver fibrosis, the exact mechanisms by which liver fibrosis occurs and progresses are complex and may involve a multitude of factors [4]–[10]. Recently, much interest has been re-focused on the potential role that lipopolysaccharide (LPS) could play in the development of liver fibrosis [11]–[13]. LPS has long been associated with liver fibrosis and has been an accepted agent to induce fibrosis in animal models [12], [14]. Measurable levels of LPS can be detected in the serum or plasma of healthy individuals and they are thought to arise from the continual exposure to products from the enterobacteria [12], [14]. Under normal conditions, low levels of LPS are effectively neutralized via several peripheral proteins such as LPS binding protein (LBP) and soluble CD14 (sCD14) [15]. LPS is further modified enzymatically via acyloxyacyl hydrolase, an enzyme that deacylates LPS and effectively prevents its ability to activate the TLR4 pathway [16].

Using comparative glycoproteomics we have determined that the glycosylation of IgG molecules reactive to the heterophilic anti-gal epitope increase with the development of liver cirrhosis [17]. Heterophilic anti-gal antibodies are naturally occurring antibodies that constitute ∼1% of total serum immunoglobulin and interact with a specific sugar linkage on glycoproteins and glycolipids such as LPS [18]–[20]. This sugar linkage (Gal α-1-3Galβ1-(3)4GlcNAc-R), referred to as the alpha-gal epitope, is absent in humans but is abundantly synthesized by bacteria, nonprimate mammals, and New World monkeys. It has long been believed that anti-gal antibodies control the level of *Enterobacteriaceae*, which are commonly found as a normal part of the human and animal gut flora and express the alpha-gal epitope [18].

In this report we show that the glycosylation of anti-gal antibodies are altered in patients with HCV induced liver fibrosis and importantly, the ability of these modified antibodies to lyse target cells are dramatically reduced and may contribute to endotoxin exposure in people infected with HCV. In addition, we show that similar to what we have previously observed in patients co-infected with human immunodeficiency virus (HIV) and HCV [21], patients infected with HCV have increased levels of markers of microbial translocation.

## Results

### Alpha-gal Antibodies are Altered in Amount and Type of Glycosylation in Patients with Liver Fibrosis and Cirrhosis

**Figure 1:**
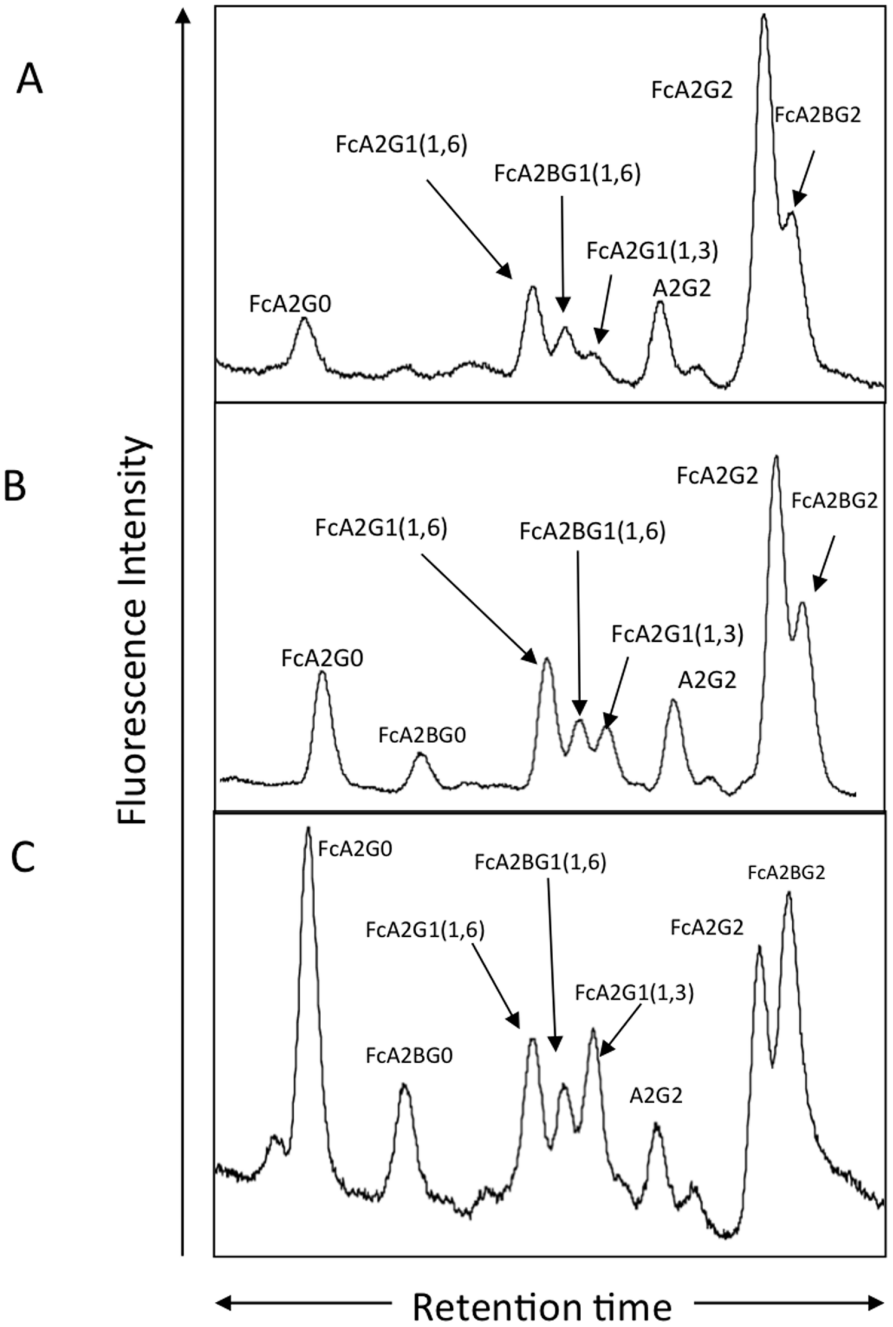
The glycosylation of anti-gal IgG is altered with the development of fibrosis and cirrhosis. Anti-gal IgG was purified from a pool (n = 20) of healthy individuals, pooled ( = 20) fibrotic individuals (stage 1–2) or pooled (n = 20) late stage fibrotic (cirrhotic) individuals (stage 5–6). Glycan analysis of the N-linked glycans associated with the heavy chain from the healthy individuals (panel A), the fibrotic individuals (panel B) or the late stage fibrotic individuals (panel C). For structures presented in panels A-C: FcA2G0, core fucosylated (1,6) agalactosylated biantennary glycan; FcA2G0B, core fucosylated (1,6) agalactosylated biantennary glycan with a bisecting N-acetylglucosamine (GlcNac); FcA2G1 (1,6) core fucosylated (1,6) biantennary glycan with a single galactose residue on the 1,6 arm; FcA2G1B (1,6) core fucosylated (1,6) biantennary glycan with a single galactose residue on the 1,6 arm and a bisecting GlcNac; FcA2G1 (1,3), core fucosylated (1,6) biantennary glycan with a single galactose residue on the 1,3 arm; FcA2G2, core fucosylated biantennary N-glycan (FcA2G2); FcA2G2B, bisected core fucosylated biantennary N-glycan.

Patients for the current analysis were obtained from the University of Michigan and St. Louis University School of Medicine under a study protocol that was approved by the University’s Institutional Review Board. Demographic and clinical information was obtained, and a blood sample was collected from each subject (summarized in Table 1). Recently we have determined that the N-glycosylation of IgG molecules that recognize the heterophilic alpha-gal epitope (Gal α-1-3Galβ1-(3)4GlcNAc-R) changes with the development of liver cirrhosis [17]. Specifically, the change in glycosylation on anti-gal IgG molecules is a decrease in the level of galactosylation leading to anti-gal IgG molecules containing N-linked glycans devoid of galactose residues. We have extended this work and show that this loss is observed even in patients with early fibrosis (Stage 1–2 ISHAK) (Figure 1), as well as people with more advanced fibrosis (Stage 5–6 ISHAK) (Figure 1C). As Figure 1A shows, while anti-gal antibodies from healthy patients have 41% (±1.6%) of the fully galactosylated glycan, patients with fibrosis (Figure 1B) have 31% (±3.9%) and patients with cirrhosis (Figure 1C) have only 13% (±2.3%). This change in glycosylation was detectable by an increased reactivity with fucose binding lectins and allowed for the development of a plate based assay to measure this change [17]. In this plate based assay, anti-gal immunoglobulin is captured with an alpha gal containing antigen (Galα1-3Galß1-3GlcNAc conjugated to human serum albumin) and the glycosylation determined using biotinylated lectins.

**Table 1:** Sample population characteristics.

Obtained From:	University of Michigan^1^			St. Louis University^1^	
Disease Diagnosis^2^:	Controls	Stage 1–2 fibrosis	Stage 5–6 (Cirrhosis)	Controls	Stage 5–6 (Cirrhosis)
Number	20	21	19	21	20
Etiology% (HBV/HCV/crypto/alcohol/other)^3^	–	0/100/0/0/0	85/11/2/2	11/51/24/10/4	5/48/20/18/9
Age	58.04±11	50±8	53±8	58.6±12	58±3
ALT (IU/mL)^4^	N/A	75±7	79±19	N/A	N/A
AST (IU/mL)^5^	N/A	73±5	93±11	N/A	N/A
Total Bilirubin^6^ (mg/dL)	N/A	0.3±0.2	1.2±1.3	N/A	N/A
Gender M:F%	71∶29	84∶16	80∶20	75/25	60/40
MELD Score^7^	N/A	N/A	10.3±4	N/A	9±2
Child Class (A/B/C/) or NA%^8^	–	–	–N/A	–	40/54/6/0

1 Samples were provided from St. Louis University Medical School or from the University of Michigan. See text for more details.

2 Disease diagnosis was determined by MRI or by liver biopsy.

3 For Etiology: HBV, hepatitis B virus; HCV, hepatitis C virus; crypto, cryptogenic liver disease; alcohol, alcohol induced liver disease; other, liver disease of unknown origin.

4 ALT, Alanine transaminase.

5 AST, aspartate aminotransferase.

6 Total Bilirubin levels.

7 MELD: Model for end stag liver disease. N/A, not available.

8 The percent of patients with each Child-Pugh score is given as a percentage in each group.

**Figure 2:**
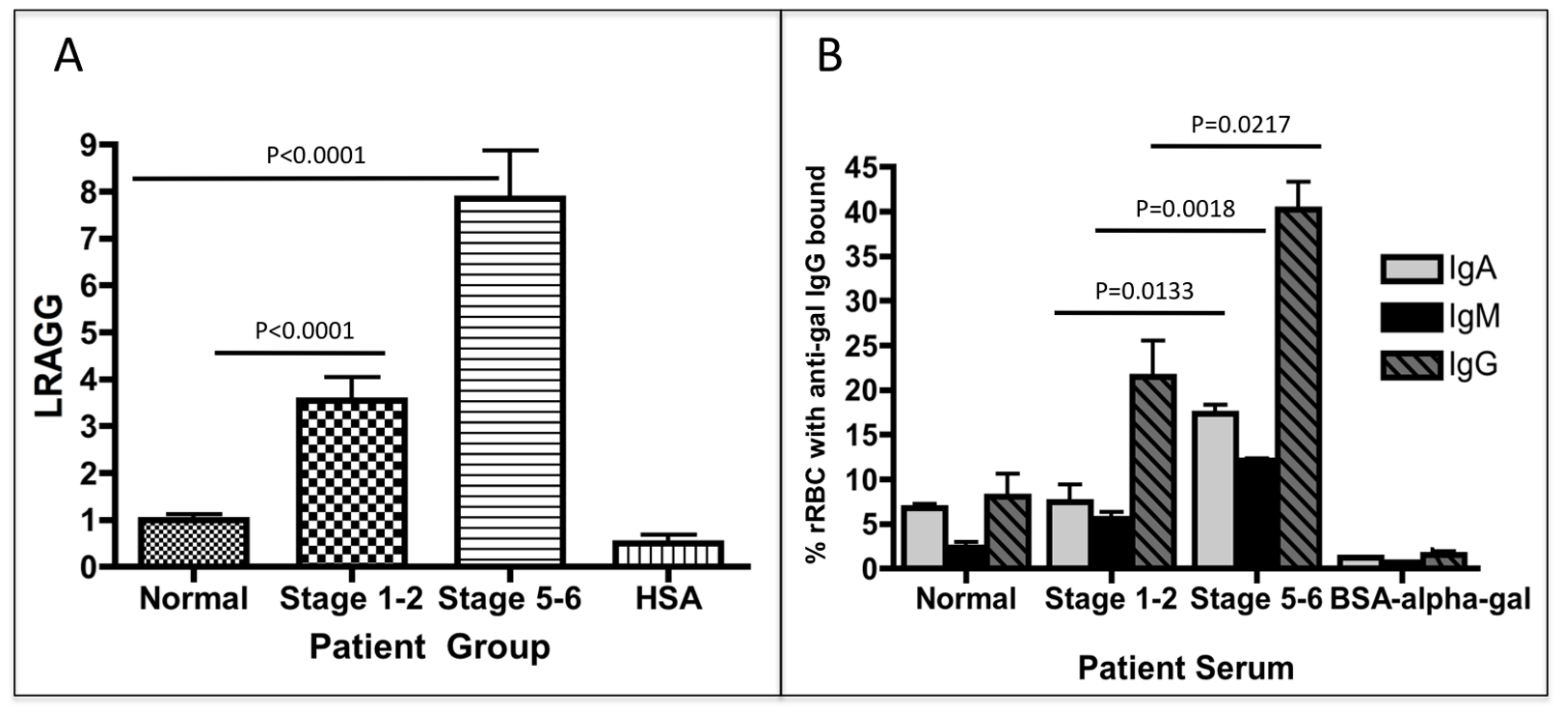
Increased lectin reactive anti-gal IgG from patients with increasing levels of liver fibrosis. A) Compared to commercially purchased normal human sera, patients with limited liver fibrosis (stage 1–2) have a 3.5 (±1.4) fold increase in lectin reactive anti-gal IgG (LRAGG). More advanced (Stage 5–6) fibrosis patients have a 7.9 (±2.4) fold increase in LRAGG. The fold increase in LRAGG is statistically significant (P<0.001). When serum from more advanced fibrotic patients is used on plates coated with HSA alone and not HSA-alpha-gal, no signal is observed (HSA lane). B) The level of anti-gal IgA, IgM and IgG bound to target rabbit red blood cells as a function of liver fibrosis. There is a statistically significant increase in anti-gal IgA from commercially purchased normal human serum to serum from patients with advanced fibrosis (P = 0.0048); Anti-gal IgA also significantly increases from limited to advanced fibrosis (P = 0.0133). Anti-gal IgM significantly increases from control to limited fibrosis (P = 0.02) and from control to advanced fibrosis (P = 0.002); there is also a significant increase in anti-gal IgM from limited to advanced fibrosis (P = 0.0018). Anti-gal IgG is significantly elevated in advanced fibrosis compared to control (P = 0.0075) and also from limited to advanced fibrosis (P = 0.0217). 50 mM of BSA-alpha-gal can prevent binding of antibodies to RBCC. See text for more details. For panels A & B, samples size is Normal, n = 21; Stage 1–2, n = 22; and Cirrhosis, n = 39.

As shown in Figure 2A this plate-based lectin assay is able to detect changes in glycosylation with the progression of liver disease. Specifically, patients with mild fibrosis (Stage 1–2 ISHAK; n = 21) have a 3.5 (±1.4) fold increase in lectin-reactive anti-gal immunoglobulin (LRAGG) over normal serum (n = 20), while patients with more advanced fibrosis (ISHAK 5–6) n = 39) have a 7.9 (±2.4) increase in LRAGG. In Figure 2B we show that, concurrent with the change in glycosylation, there is a change in the amount of anti-gal immunoglobulin in patients with liver disease. Figure 2B shows an increase in levels of anti-gal IgA, IgM and IgG bound to target rabbit red blood cells as a function of liver fibrosis. As shown, there is 3 fold increase in anti-gal IgG, in patients with Stage 1–2 fibrosis as compared to normal serum and close to a five fold increase in anti-gal IgA, IgG and IgM in patients with Stage 5–6 fibrosis (cirrhosis). To determine the specificity of the interaction we demonstrated that the binding of antibodies to rRBC could be completely abolished by the addition of the sugar Gal α-1-3Galß1-(3)4-GlcNAc, which brought immunoglobulin binding of antibodies to background levels (labeled as BSA-alpha-gal). In contrast addition of the disaccharide lactose had little effect on IgG binding to the rRBC (data not shown).

### Alpha-gal Antibodies with Altered Glycan have Reduced Complement Mediated Killing Ability

**Figure 3:**
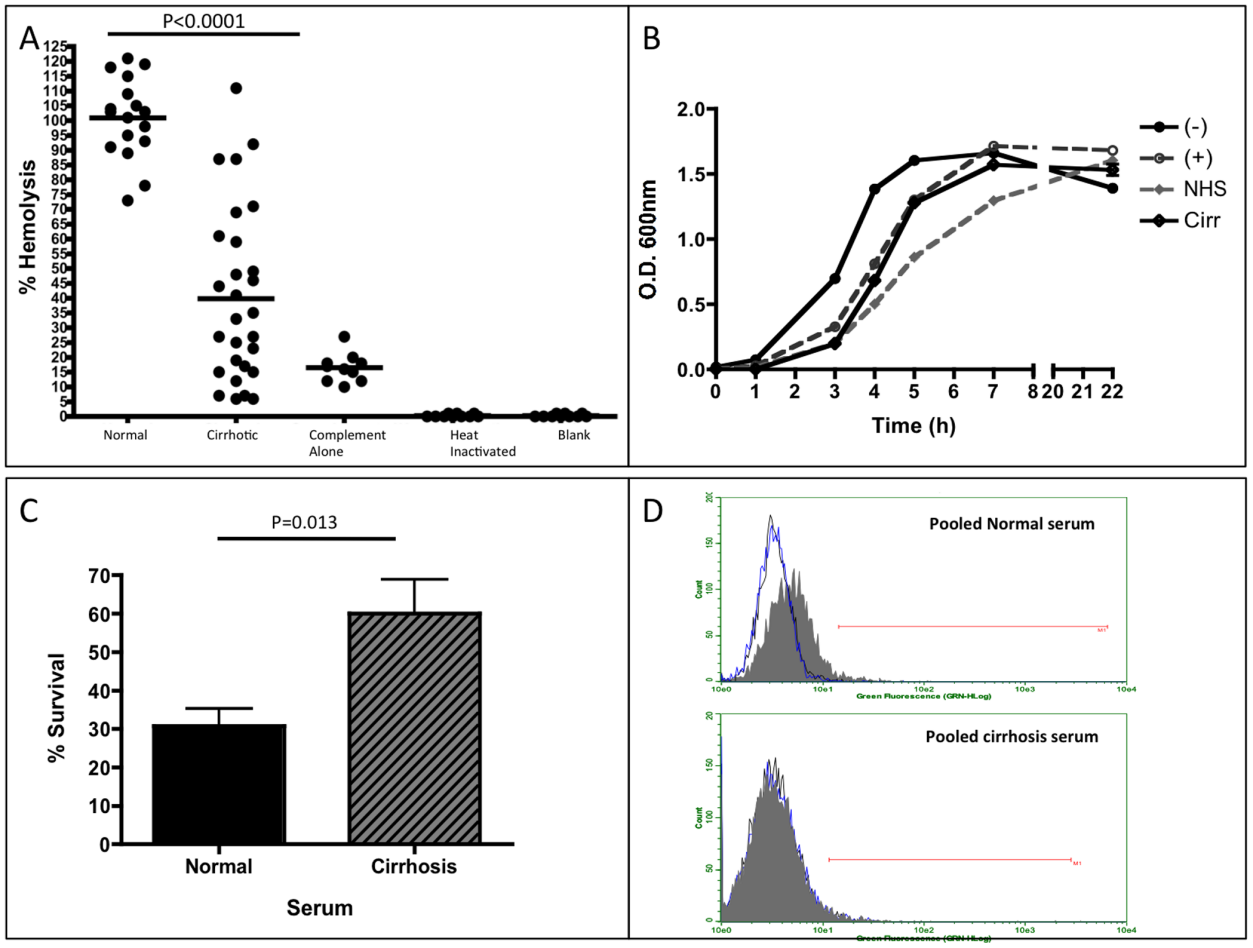
Anti-gal specific antibodies have poor complement mediated killing ability and are unable to induce phagocytosis of opsonized target cells. A) Results from a hemoglobin release assay using serum from control patients and patients with cirrhosis. Compared to normal serum, serum from cirrhosis patients has an over 60% decrease in the capacity to induce complement mediated killing of target rRBCs. For panel A, sample size is: Normal, n = 20 and Cirrhotics, n = 20. As a control, complement alone was used to indicate the level of the alternative pathway (complement alone). In addition, if normal samples were heat inactivated or not treated with serum or complement, no lysis was observed. B) Results from a bactericidal assay using human serum show the growth pattern of bacteria alone (–), bacteria incubated with functional complement (+), bacteria with functional complement and normal human serum (NHS), or bacteria with functional complement and serum from a pool of 20 cirrhosis patients (Cirr). Error bars are indicated. C) Bacteria incubated with serum from a pool of 20 cirrhosis patients, in the presence of functional complement, show a significantly increased survival rate compared to those exposed to normal human serum (P = 0.013). Data normalized to those that did not receive serum addition. D) Results from an opsonization/phagocytosis assay. Bottom panel show target cells opsonized with serum from cirrhosis patients are not phagocytosed by monocytes, while top panel shows target cells opsonized with purchased normal serum are phagocytosed. Black peak represents monocytes alone, blue peak represents monocytes incubated with non-opsonized target cells, and gray filled peak represents opsonized target cells incubated with monocytes.

As recent reports have indicated that the ability of IgG to bind and activate complement molecules is dependent upon the type of N-linked glycan attached [22], we examined the ability of anti-gal antibodies from patients with liver cirrhosis to bind and activate exogenously added complement. It is important to note that this assay is designed to determine how well the antibodies with different glycosylation can bind and activate exogenously (experimentally) added complement and is not affected by the levels of complement that may be present (or not) in each individual. Briefly, rabbit red blood cells (rRBC) were incubated with heat inactivated serum (to destroy any residual complement activity) from patients with varying levels of liver disease and supplemented with functional human complement as described previously [23]. Thus, each sample receives an equal amount of complement and the difference in anti-gal immunoglobulin is the only variable in the assay. Cell lysis is measured by the release of hemoglobin. Figure 3A shows the results of the rRBC lysis assay using serum from control patients (n = 20) and from patients with liver cirrhosis (n = 20). Heat inactivated patient serum without the addition of complement had no killing activity (See figure 3A). In contrast, when functional human complement was added to heat inactivated serum, a clear and statistically relevant pattern emerges. As shown in Figure 3A, alpha gal specific immunoglobulin from control patients had similar levels of killing (mean of 100.9% ±13.63%) as compared to the commercially normal human serum (normalized to 100%). In contrast, the level of complement mediated killing was decreased in patients with liver cirrhosis with a mean of 39.8% killing (±29.3%) as compared to the level observed in commercially normal human serum (*p*<0.0001). As a control, complement alone was used to indicate the level of killing via the alternative pathway (complement alone). In addition, when normal samples were heat inactivated or left untreated with serum or complement (blank), no lysis was observed.

### The Serum from Patients with Liver Cirrhosis have Altered Bactericidal Activity

There is an established body of evidence suggesting that alpha-gal specific antibodies are reactive towards the Enterobacteriaceae, which are commonly found as a normal part of the human and animal gut flora and express the alpha-gal epitope [19], [24]. As our results showed there was a decrease in the complement mediated killing of rRBC in an anti-gal dependent manner, we next examined the influence of the anti-gal molecules from patients with early fibrosis and those with late fibrosis in their ability to effect bacterial growth. Briefly, a strain of E. coli. (O86:B7) that has been shown to bind alpha gal antibodies [25] was incubated over night and the next day a fixed amount of the bacterial suspension was incubated with functional complement and supplemented either with heat inactivated serum from pooled cirrhotic patients (20 patients/pool) or heat inactivated normal serum. Figure 3B shows the pattern of bacterial growth in the presence of serum. Compared to bacteria alone (labeled as –), bacteria incubated in the presence of exogenous complement (labeled as +) showed a delayed capacity for growth. The addition of normal serum further impeded bacterial growth over time (labeled as NHS), whereas the addition of pooled cirrhotic serum had no additive effect in the presence of exogenous complement (labeled as cirr). The addition of purified non-specific immunoglobulins containing terminal galactose, such as that found in healthy patients had no effect on bacterial growth in the presence of functional complement; however, the addition of purified agalactosylated IgG impeded bacterial growth similar to that shown with patient sera (data not shown). We were unable to perform similar analysis with immunoglobulin from patients with cirrhosis due to sample limitations.

In Figure 3C we show that the serum from cirrhosis patients had reduced bacterial killing in the presence of functional complement, as compared to the serum from healthy controls. That is, compared to the cultures with the addition of normal serum, the addition of cirrhosis serum resulted in roughly a 100% increase in bacterial survival (from 30% to close to 60%). Briefly, bacteria 1 hour after growth (as in 3B) were plated on nutrient agar plates and allowed to form colonies overnight. The number of colonies from cultures incubated with pooled cirrhotic serum was increased by close to 100% as compared to the control serum. Consistent with the data in Figures 3 A & B, this result strongly suggests that the serum, and most likely the antibodies from the patients with cirrhosis have less anti-bacterial neutralizing ability than healthy patients.

To complete our investigation of the function of anti-gal IgG, we examined the ability of THP-1 monocytes to phagocytose target red blood cells opsonized with normal serum or serum from patients with cirrhosis. Briefly, rRBC were labeled with a GFP reagent that freely penetrates the cell membrane, interacts with intracellular glutathione transferase and results in stable cytoplasmic staining. The labeled rRBCs were then washed and opsonized with heat-inactivated serum. The opsonized rRBCs were washed again and then incubated overnight with THP-1 monocytes. The monocytes were fixed, and counter-stained with an anti-CD11c-PE conjugated antibody and analyzed by flow cytometry. The black peak represents monocytes alone, blue peak represents monocytes incubated with non-opsonized target cells and gray filled peak represents opsonized target cells incubated with monocytes.

As expected, monocytes were able to phagocytose target rRBCs opsonized with normal serum (Figure 3D, top panel), as shown by a shift in the grey peak. However, there was no change in the phagocytosis of target rRBCs incubated with serum from cirrhosis patients (Figure 3D, bottom panel).

### Peripheral Markers of LPS Exposure in Patients with Liver Fibrosis/cirrhosis Correlate with the Level of Glycan Modified Anti-gal Antibodies

**Figure 4:**
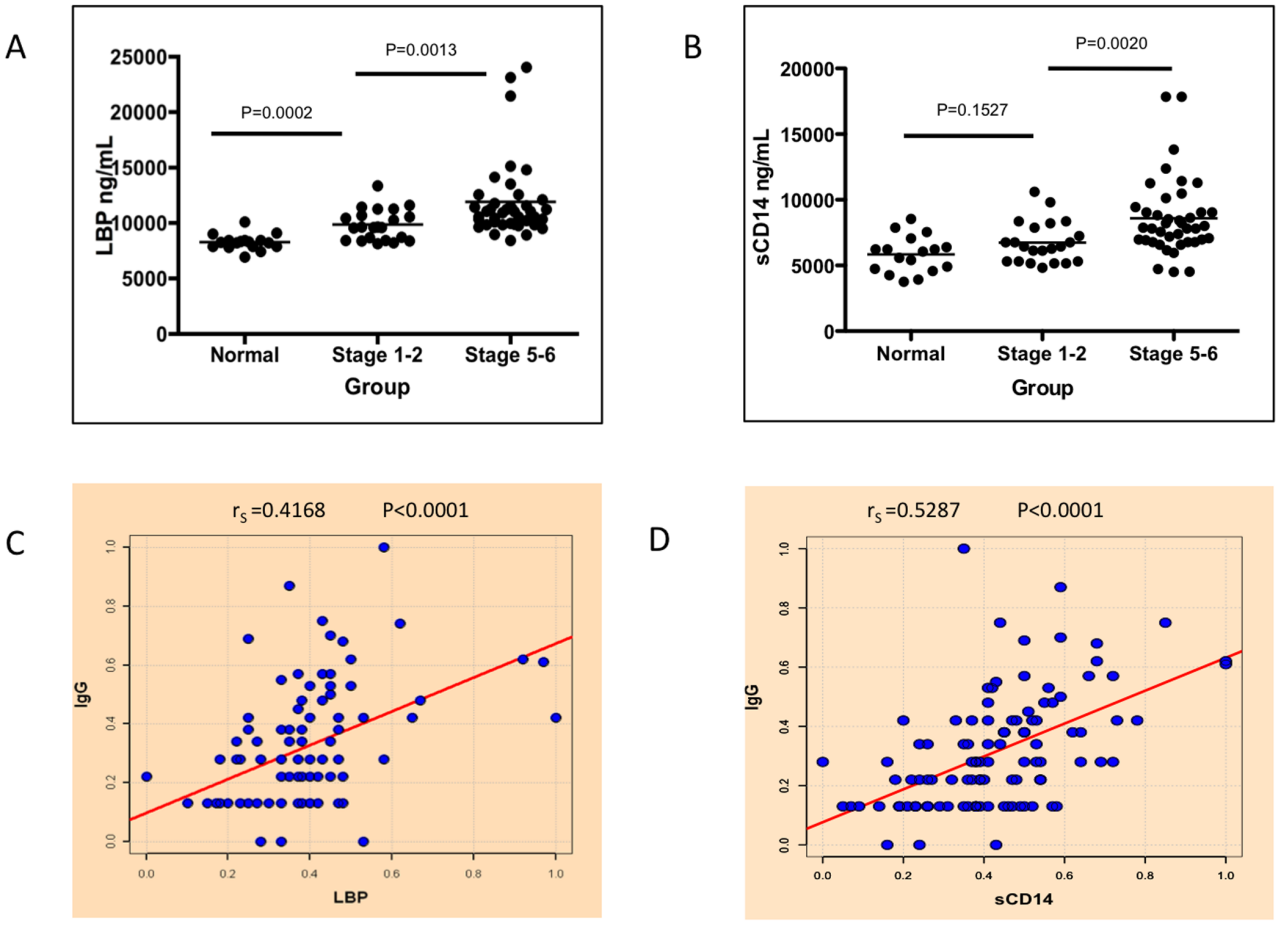
Peripheral markers of LPS exposure in patients with liver fibrosis correlate with the level of LRAGG. A) There is a statistically significant increase in the level of LPS Binding Protein (LBP) detected in the sera of patients with mild liver fibrosis (P = 0.0002) and also as a function of liver cirrhosis (P = 0.0013). B) A similar increase is seen in soluble CD14 (sCD14) with the progression of liver fibrosis to liver cirrhosis (P = 0.0020). C) There is a direct correlation between LBP and LRAGG (r_S_ = 0.4168, P<0.0001) and between sCD14 and LRAGG (r_S_ = 0.5287, P<0.0001) (D). r_S_ denotes the Spearman’s correlation coefficient. For panels A & B, samples size is Normal, n = 20; Stage 1–2, n = 21; and Stage 5–6 (Cirrhosis), n = 39.

Since we have observed that patients with more advanced fibrosis show a reduced killing of alpha-gal containing target cells (Figure 3A) and actually inhibited complement mediated killing of bacteria (Figure 3B–C), it was of interest to see if these same people also had evidence of bacterial exposure in their circulation. We have done this through the measurements of markers of endotoxin exposure such as soluble CD14 (sCD14) and LPS binding protein (LBP) in patients with varying levels of liver fibrosis. Both of these proteins are upregulated in the presence of LPS and have been used by us and by others as markers of bacterial exposure [21]. Briefly, the levels of LBP and sCD14 were determined in patients with varying levels of liver fibrosis through the use of a commercially available anti-sCD14 and LBP ELISA kits. As Figure 4A shows, while all patients have basal levels of LBP (panel A) or sCD14 (panel B), patients with increasing levels of liver fibrosis have much greater levels of circulating sCD14 and LBP, suggesting that these patients may have increasing levels of LPS in their circulation. That is, normal patients (n = 21) have a mean of 8289 ng/ml (±702) of peripheral LBP, patients with limited fibrosis (n = 21) have 9836 ng/mL (±1382) and patients with advanced fibrosis/cirrhosis (n = 39) have 11877 ng/mL (±3471). A similar increase is observed with sCD14, where normal patients have a mean of 5843 ng/ml (±1394) of peripheral sCD14, patients with limited fibrosis have 6744 ng/mL (±1577) and patients with advanced fibrosis have 8599 ng/mL (±2913). For both LBP and sCD14, there was a statistically significant difference between the fibrosis groups (p<0.05; see figure 3). These data suggest that HCV patients, like HIV/HCV co-infected patients, may have increased levels of LPS exposure early in their liver fibrosis. Figures 4C–D show the relationship between the level of these peripheral markers of endotoxin exposure and the level of LRAGG. As this figure shows, there is a direct correlation between the level of LRAGG and the peripheral markers of endotoxin exposure. LRAGG was correlated both to LBP with a Spearman's correlation coefficient of 0.4168 (P<0.0001) and sCD14 with a Spearman's correlation coefficient of 0.5287 (P<0.0001). LPB and sCD14 also correlated with each other with a Spearman's correlation coefficient of 0.4665 (P<0.0001).

### Peripheral Markers of LPS Exposure in Patients with Liver Fibrosis and Cirrhosis Change in Response to Interferon Treatment

Consistent with the work in vitro, and with our work with HIV/HCV co-infected individuals [21], there was indeed an increase in peripheral markers of bacterial exposure in people with liver fibrosis and this correlated with the levels of anti-gal antibodies with modified N-linked glycan. Next, we wanted to determine if patients with liver disease who were successfully treated and cleared of their viral infection, saw a change in the markers of bacterial exposure. A total of 108 HCV mono-infected individuals were enlisted in the study. There were 41 who responded to a course of interferon-alpha therapy (Responders), while 67 still had active viral disease following treatment (Non-responders). Available demographic and clinical data are summarized in Table 2. Patients were well matched in age with a mean of 44.9 years for the Responders group and 44.2 years for the Non-responders group. Likewise, patients were matched for their severity of liver disease in both grade of inflammation and fibrosis stage. Patients had a mean Knodell score of 2.4 and 2.3 in the Responder and Non-responder groups, respectively. Metavir scoring indicated the mean stage of fibrosis was 2.6 (Responders) and 2.5 (Non-responders).

**Table 2:** Patient Samples Utilized in Study.

Treatment Group	N	Diagnosis	Active DiseaseFollowing Treatment	Mean Age	Gender(% Male)	Mean Grade ofInflammation	Mean Stage of Fibrosis
Responders	41	HCV	No	44.9	58.5	2.4	2.6
Non-responders	67	HCV	Yes	44.2	70.1	2.3	2.5

a Samples were provided coded from St. Louis University Medical School.

b Grade of inflammation was determined at the time of liver biopsy, characterized by histologic activity index (HAI) and given a corresponding Knodell score.

c Fibrosis staging done at time of liver biopsy using Metavir scoring system.

**Figure 5:**
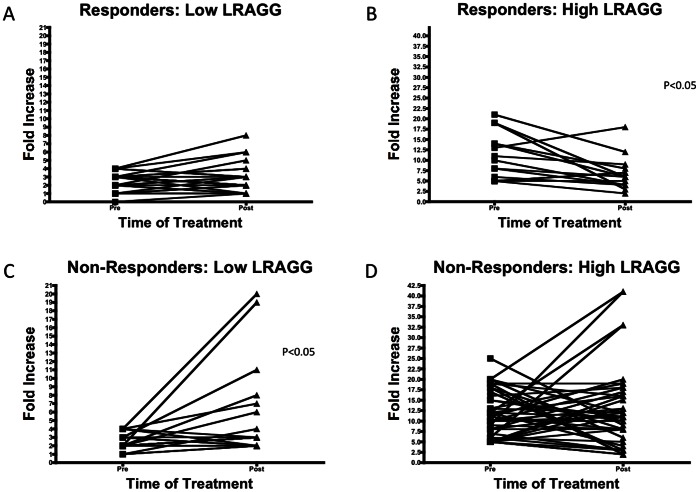
Change in LRAGG in response to IFN Treatment in patients with liver fibrosis. (A) The change in the level of LRAGG in patients who responded to IFN and cleared their HCV and had low LRAGG at start of treatment. As this figure shows, there is no significant change in patients who started with low levels of LRAGG in response to IFN therapy. (B) In contrast, those patients who responded to IFN and cleared their HCV and started had greater than 5-fold increase over normal at the start of treatment show a significant decrease following treatment (P<0.05) (C). However, patients who do not respond to IFN treatment show a significant increase (P<0.05) following treatment when they began with low levels (C) and stayed high when they started with greater than 5-fold increase in LRAGG over normal (D).

We then determined the level of LRAGG in these patients. In this case we found it was of great interest to determine the pattern of changes in the fold increase or decrease in LRAGG in those patients who began a treatment course with either low or high levels of LRAGG. Figure 5A shows the fold increase in LRAGG in Responders who started treatment with low levels of LRAGG (defined as less than a 5-fold increase over commercial sera). As Figure 5A shows there is no significant change in LRAGG following the course of treatment; in other words those who started low stayed low. However, Figure 5B shows a significant decrease in LRAGG in Responder patients who started treatment with high LRAGG levels (defined as 5-fold or greater increase over commercial sera) (P<0.05). The pattern was reversed in patients in the Non-responder group; they saw a significant increase in LRAGG (P<0.05) when they started therapy with low levels (Figure 5C) and no significant overall change when they started with high LRAGG (Figure 5D).

**Figure 6:**
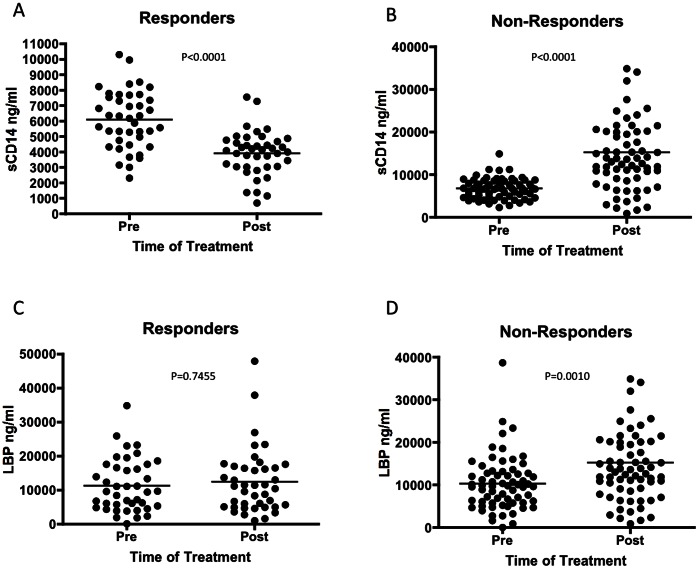
Markers of bacterial exposure change in response to IFN therapy in patients with liver fibrosis. Patient sera were tested for sCD14 and LBP levels pre- and post- IFN therapy. In patients who respond to treatment, there is a statistically significant decrease in sCD14 following treatment (P<0.0001) while patients who do not respond show a significant increase following the course of treatment (P<0.0001) (B). There was no significant change LBP between treatment groups in patients who respond to IFN therapy (C), however patients who do not respond to treatment show a statistically significant increase from pre-treatment LBP levels (P<0.001) (D).

In Figure 6, we examined the levels of peripheral markers of bacterial exposure in these patients and demonstrate that patients in the Responder group showed a significant decrease in sCD14 from 6088 ng/ml to 3905 ng/ml in response to IFN-therapy (P<0.0001) (Figure 6A). Conversely, in Non-responders the mean sCD14 increased from 6735 to 15244 ng/ml following treatment (Figure 6B).

Additionally, those who respond successfully to treatment showed no change in the level of LBP detected in response to treatment with a mean of 11248 ng/ml pre-treatment to 12429 ng/ml post-treatment (Figure 6C). Patients who did not respond to treatment showed a statistically significant increase in LBP post-treatment from a mean of 10315 to 15244 ng/ml (Figure 6D, P<0.001).

## Discussion

In our initial study, we had discovered an antibody that was altered in both amount and in glycosylation as a function of liver fibrosis regardless of the etiology of liver disease [17], [26]. In this study we have analyzed the functionality of these immunoglobulins and have discovered that, surprisingly, they have dramatically reduced complement dependent killing against both alpha-gal containing rRBCs and against bacteria. It is important to note that we are unclear if there is a selective increase in agalactosylated anti-gal IgG in patients with liver fibrosis/cirrhosis or if other immunoglobulin molecules are also altered functionally.

Based upon these results, we wanted to know if, in people with increasing amounts of anti-gal IgG, there was also an increase in the amount of peripheral markers of bacterial exposure. Consistent with the work in vitro, with our work with HIV/HCV co-infected individuals [21], and with work by others [27], [28], there was indeed an increase in peripheral markers of bacterial exposure in people with liver fibrosis and this correlated with the levels of anti-gal antibodies with modified N-linked glycan. This suggests that liver function, as influenced by the presence of HCV, plays a role in the presence of microbial products in the circulation and the generation of the antibody response.

One question that remains is why there is a reduction in the complement mediated lysis reaction from patients with cirrhosis. As these results could be the consequence of a shift from an IgM to IgG responses, the level of anti-gal reactive IgM was measured and found to increase in patients with more severe liver disease (Figure 2B). This raises an interesting point: If there are increased amounts of IgM bound to rRBC in patients with cirrhosis, why don’t anti-gal IgM’s from the cirrhotic patients induce the same degree of cell lysis? IgM can easily activate complement and should mediate lysis. It is possible that the 30% of the lytic activity seen in the cirrhotics is due to IgM, with the remaining killing activity coming from anti-gal IgG and IgA. It is noted in our analysis, and consistent with published results [19], [20], [24], [29], the anti-gal IgG from all patients is primarily of the IgG2 subclass (data not shown), which is not efficient at binding and activating the classical complement pathway. Hence, it could be a decrease in the ability of the anti-gal IgG to activate the alternative complement pathway. However, reports in the literature have shown that, for some antibodies, the change in the glycosylation that we observe on anti-gal IgG (and potentially IgA) is actually associated with greater binding to the mannose binding lectin (MBL) and greater activation of the complement pathway as opposed to the decrease that we have observed (and here) [30], [31]. It is also noted that reductions in complement have been observed in patients with liver cirrhosis [32], implying that the results presented here could be much greater in patients with liver disease. Additionally, an explanation for the results presented in figure 3 is the presence of are other non-heat labile, non-antibody factors, in the cirrhotic subject serum that enhanced bacterial growth during culture.

Several reports have examined the role of anti-gal antibodies in relation to the gut *Enterobacteriaceae* and in the rejection of xenotransplantation [18], [23], [33], [34]. One study indicated that anti-gal antibodies can actually prevent the complement mediated killing of target bacteria and may actually aid in the survival of bacteria in the bloodstream [24]. In regards to the immune attack of xenotransplants, reports have indicated that certain anti-gal IgG molecules can inhibit the complement mediated lysis of target cells via anti-gal IgM molecules [35], [36].

In conclusion, the results presented indicate that the generation of an antibody response to bacterial products may actually be pathogenic through increasing exposure to endotoxin. It is also noted that anti-gal IgG may be a potential agent for enhancing bacterial exposure in people with liver disease that could be a target for therapeutic intervention.

## Materials and Methods

### Patients

Patients for the current analysis were obtained from the University of Michigan (20 control patients, 21 patients with stage 1–2 fibrosis (Ishak) and 19 patients with stage 5–6 fibrosis (Ishak) and St. Louis University School of Medicine (21 control patients without any liver disease, 20 patients with cirrhosis. In addition, a second set of 108 patients with stage 2–3 fibrosis (metavir) who were treated with interferon were obtained from St. Louis University School of Medicine. In all cases samples were collected under a study protocol that was approved by the University’s Institutional Review Board. In addition, written informed consent was obtained from each subject. Demographic and clinical information was obtained, and a blood sample was collected from each subject (summarized in Table 1). The blood sample from patients with chronic HCV infection was obtained at the time of liver biopsy and antiviral therapy. HCV was defined as the presence of HCV RNA with a lower limit of detection of <50 IU/mL. All liver biopsies were at least 30 mm long and 1.4 mm wide and graded by three hepatic pathologists in a blinded fashion and the amount of fibrosis was graded using the Ishak scoring system. A group of individuals with no history of liver disease, alcohol consumption less than 40 gm a week, and no risk factors for viral hepatitis were enrolled from the General Internal Medicine clinics. All subjects in this control group were documented to have normal liver biochemistry and negative HCV antibodies.

### Purification and Glycan Analysis of Anti-gal IgG

For analysis of anti-gal IgG, pooled serum from 10 healthy controls, 10 patients with stage 1–2 fibrosis (Ishak) and 10 patients with cirrhosis were used. 2 µl from each patient for a total of 20 µl in each pool was utilized. Briefly, synthetic Galα1-3Galß1-3GlcNAc-HSA (Dextra Labs, Reading, United Kingdom) was coupled to a NHS-activated Sepharose 4 Fast Flow affinity column (GE Healthcare, Piscataway, NJ) as per manufacturer’s directions. 20 µl of serum was incubated with the column and the column washed with five column volumes of TBS-T (Tris buffered saline with 0.1% Tween-20), followed by one column volume was with TBS. The Galα1-3Galß1-3GlcNAc specific IgG was eluted using 0.1M NaCl_2_/0.1M Glycine pH 2.8 and immediately neutralized. For this study, an equal amount of anti-gal IgG (1 µg) was reduced, alkylated and separated on a 12% tris-glycine acrylamide gel and stained using Colloidal Coomassie. Anti-gal IgG bands were excised, destained and glycan analysis preformed as done previously and as described above [37], [38]. For quantification, area under the curve (AUC) values for were determined by the Waters Millennium software the %peak volume for each peak. The total % of all peaks identified was 100%. This is similar to what has previously been done [39]–[43].

### Lectin Fluorophore-Linked Immunosorbent Assay (FLISA)

For the plated based analysis of the glycan modification of anti-gal IgG, we utilized a lectin-FLISA based approach [17]. Briefly human serum albumin attached to Galα1-3Galβ1-3GlcNAc (HSA-alpha-gal; Dextra Labs) or HSA alone (Sigma-Aldrich), was added to the plate and following incubation overnight washed with 0.1% Tween 20/PBS 7.4 and blocked overnight with 3% BSA/PBS. For analysis, 3 µl of serum was diluted in 97 µL of 3%BSA/PBS and added to the plates for 2 hours and washed 5 times in lectin incubation buffer (10 mM Tris pH 8.0, 0.15 M NaCl, 0.1% Tween 20) before fucosylated IgG detected with a biotin conjugated Aleuria aurantia (AAL) lectin (Vector Laboratories, Burlingame, CA). Bound lectin was detected using IRDye™ 800 Conjugated streptavidin and signal intensity measured using the Odyssey® Infrared Imaging System (LI-COR Biotechnology, Lincoln, Nebraska). In all cases sample intensity was compared to commercially purchased human serum (Sigma Inc., St Louis, MO.). All samples were run in triplicate and inter sample variation was less than 1%.

### FACS Analysis of Anti-gal IgA, IgM, and IgG Binding to Rabbit Erythrocytes

Approximately 8.5×10^7^ rabbit RBCs (rRBCs) per sample were washed in 1X HBSS. The rRBCs were resuspended to a final volume of 50 ul per sample and incubated at 37°C for 1 h with heat-inactivated purchased normal serum or a composite of patient sera at 1∶10 dilution as indicated. Incubations were carried out in U-bottom 96-well plates to facilitate processing of multiple replicates. Following incubation, the cells were pelleted with gentle centrifugation (1500 rpm, 1 minute) and washed twice with 1X HBSS. In order to determine binding of the anti-gal antibodies to the alpha-gal epitope of the rRBCs, Goat-α-human IgA-FITC, IgG-FITC, and IgM-PE (Southern Biotech, Birmingham AL) were added for one hour at 37°C. Goat-α-human IgA-FITC and Goat-α-human IgG-FITC were added at 1∶400 and Goat-α-human IgM-PE was added at 1∶800 in HBSS. Cells were again washed after incubation and analyzed by flow cytometry using the Guava Flow Cytometry System (Millipore, Billerica, MA).

### Hemoglobin Release Assays

Hemolysis assays were performed at 37°C using approximately 8×10^6^ rabbit red blood cells (Lampire Biologicals, Pipersville, PA) in a 100 ul volume. Cells were washed in HBSS and incubated with heat inactivated (30 min at 56°C) serum samples diluted 1∶20 in HBSS for 1 h. rRBCs were then washed and incubated with normal human serum minus IgA, IgM and IgG (Sigma, St. Louis, MO) as a source of exogenous complement, at a 1∶5 dilution for 30 minutes. Cells were gently centrifuged at 1500 rpm for 1 minute at 4°C and supernatants were transferred to a 96-well plate and read in a spectrophotometer at 541 nm. Dilutions of serum altered the degree of lysis but did not alter the relative differences in lysis.

### Bactericidal Assay

A strain of *E. coli.*, O86:B7, (ATCC) that has been shown to bind alpha gal antibodies [25]was incubated over night in Difco Nutrient Broth # 3 (Becton Dickinson) on shaker, at 37°C. Next day the bacterial suspension was adjusted to an OD_600_ of 0.6. Cells were then used to inoculate 2 ml broth containing 60 µL purified baby rabbit complement (Cedarlane, Ontario, Canada) and 100 µl of commercial (normal) or a composite of cirrhotic patient serum as indicated. Broth alone was used as a negative control for bacterial growth and complement without added serum was used as a positive control for killing via the alternative complement pathway. An aliquot of the culture was read in a spectrophotomer hourly at an optical density of 600 nm. Following hourly incubations at 37°C with shaking, the bacterial suspension was diluted 1∶35000 in broth and plated overnight on 1.5% agar (MP Biomedicals InC.) in Difco Nutrient Broth # 3 plates for 18 h at 37°C. Next day bacterial colonies were counted. The number of colonies from the untreated cultures was taken as 100% survival.

### Opsonization and Phagocytosis Assays

Approximately 8×10^6^ rRBCs were washed in sterile PBS and labeled with 2 uM CellTracker GFP labeling reagent (Lonza, Switzerland) for 30 minutes at 37°C, according to the manufacturer’s recommendations. The rRBCs were then washed, and incubated with 20 µL of normal or pooled cirrhosis patient serum for an additional 30 minutes, in the dark, at 4°C. After washing, 5×10^5^ rRBCs were added to 10^5^ THP-1 monocytes in a 96-well plate and incubated overnight at 37°C with 5% CO_2_. The next day, the cells were washed, fixed on ice for 1 hour with 2% paraformaldehyde in PBS, washed, and then counter-stained with anti-CD11c-PE (BD Biosciences, Rockville, MD) for 45 minutes at 4°C. The samples were analyzed by flow cytometry using the Guava Easycyte Flow Cytometry System.

### Statistical Analysis

Descriptive statistics for stage patients were compared by scatter plots that included the outliers. All values were reported as mean values +/− standard error unless otherwise stated. As the data did not follow typical Gaussian distribution, a non-parametrical test (two-tailed, 95% confidence, Mann-Whitney Test) was used to determine statistical difference between groups. A two-tailed P-value of 0.05 was used to determine statistical significance. Correlation between markers of microbial translocation were performed by Spearmen’s analysis. All analyses were performed using GraphPad Prism (San Diego, CA, USA).

### Analysis of LBP and sCD14

Commercially available ELISA kits were used to measure serum levels of LBP (Hycult Biotechnology, The Netherlands) and soluble CD14 (sCD14; Cell Sciences, Canton, MA). Assays were at least in duplicate according to the manufacturer recommendations.

## References

[1] Alter MJ (1997) Epidemiology of hepatitis C. Hepatology. 26: 62S–65S. 10.1002/hep.510260711 9305666

[2] Hoofnagle JH (1997) Hepatitis C: the clinical spectrum of disease. Hepatology 26: 15S–20S. 9305658 10.1002/hep.510260703

[3] Di Bisceglie AM (1997) Hepatitis C and hepatocellular carcinoma. Hepatology 26: 34S–38S. 9305661 10.1002/hep.510260706

[4] Iredale JP (2007) Models of liver fibrosis: exploring the dynamic nature of inflammation and repair in a solid organ. J Clin Invest 117: 539–548. 17332881 10.1172/JCI30542 PMC1804370

[5] Friedman SL (2007) A deer in the headlights: BAMBI meets liver fibrosis. Nat Med 13: 1281–1282. 17987019 10.1038/nm1107-1281

[6] Mehal WZ, Iredale J, Friedman SL (2011) Scraping fibrosis: expressway to the core of fibrosis. Nat Med 17: 552–553. 21546973 10.1038/nm0511-552 PMC3219752

[7] Hernandez-Gea V, Friedman SL (2011) Pathogenesis of liver fibrosis. Annual review of pathology 6: 425–456. 10.1146/annurev-pathol-011110-130246 21073339

[8] Jiao J, Friedman SL, Aloman C (2009) Hepatic fibrosis. Current opinion in gastroenterology 25: 223–229. 19396960 10.1097/mog.0b013e3283279668 PMC2883289

[9] Friedman SL (2008) Hepatic fibrosis – overview. Toxicology 254: 120–129. 18662740 10.1016/j.tox.2008.06.013

[10] Friedman SL (2007) Liver fibrosis: from mechanisms to treatment. Gastroenterologie clinique et biologique 31: 812–814. 18166858 10.1016/s0399-8320(07)73970-2

[11] Friedman SL, Rockey DC, Bissell DM (2007) Hepatic fibrosis 2006: report of the Third AASLD Single Topic Conference. Hepatology 45: 242–249. 17187439 10.1002/hep.21459

[12] Nolan JP (1989) Intestinal endotoxins as mediators of hepatic injury–an idea whose time has come again. Hepatology 10: 887–891. 2680869 10.1002/hep.1840100523

[13] Jirillo E, Caccavo D, Magrone T, Piccigallo E, Amati L, et al (2002) The role of the liver in the response to LPS: experimental and clinical findings. J Endotoxin Res 8: 319–327. 12537690 10.1179/096805102125000641

[14] Nolan JP (1981) Endotoxin, reticuloendothelial function, and liver injury. Hepatology 1: 458–465. 7030906 10.1002/hep.1840010516

[15] Gioannini TL, Teghanemt A, Zhang D, Prohinar P, Levis EN, et al (2007) Endotoxin-binding proteins modulate the susceptibility of bacterial endotoxin to deacylation by acyloxyacyl hydrolase. J Biol Chem 282: 7877–7884. 17227775 10.1074/jbc.M605031200

[16] Shao B, Lu M, Katz SC, Varley AW, Hardwick J, et al (2007) A host lipase detoxifies bacterial lipopolysaccharides in the liver and spleen. J Biol Chem 282: 13726–13735. 17322564 10.1074/jbc.M609462200

[17] Mehta AS, Long RE, Comunale MA, Wang M, Rodemich L, et al (2008) Increased levels of galactose-deficient anti-Gal immunoglobulin G in the sera of hepatitis C virus-infected individuals with fibrosis and cirrhosis. J Virol 82: 1259–1270. 18045939 10.1128/JVI.01600-07 PMC2224448

[18] Galili U (1993) Evolution and pathophysiology of the human natural anti-alpha-galactosyl IgG (anti-Gal) antibody. Springer Semin Immunopathol 15: 155–171. 7504839 10.1007/BF00201098

[19] Galili U, Anaraki F, Thall A, Hill-Black C, Radic M (1993) One percent of human circulating B lymphocytes are capable of producing the natural anti-Gal antibody. Blood 82: 2485–2493. 7691263

[20] Galili U, Rachmilewitz EA, Peleg A, Flechner I (1984) A unique natural human IgG antibody with anti-alpha-galactosyl specificity. J Exp Med 160: 1519–1531. 6491603 10.1084/jem.160.5.1519 PMC2187506

[21] Balagopal A, Philp FH, Astemborski J, Block TM, Mehta A, et al (2008) Human immunodeficiency virus-related microbial translocation and progression of hepatitis C. Gastroenterology. 135: 226–233. 10.1053/j.gastro.2008.03.022 PMC2644903 18457674

[22] Kaneko Y, Nimmerjahn F, Ravetch JV (2006) Anti-inflammatory activity of immunoglobulin G resulting from Fc sialylation. Science 313: 670–673. 16888140 10.1126/science.1129594

[23] Galili U, LaTemple DC, Radic MZ (1998) A sensitive assay for measuring alpha-Gal epitope expression on cells by a monoclonal anti-Gal antibody. Transplantation 65: 1129–1132. 9583877 10.1097/00007890-199804270-00020

[24] Hamadeh RM, Jarvis GA, Galili U, Mandrell RE, Zhou P, et al (1992) Human natural anti-Gal IgG regulates alternative complement pathway activation on bacterial surfaces. J Clin Invest 89: 1223–1235. 1556184 10.1172/JCI115706 PMC442982

[25] Posekany KJ, Pittman HK, Bradfield JF, Haisch CE, Verbanac KM (2002) Induction of cytolytic anti-Gal antibodies in alpha-1,3-galactosyltransferase gene knockout mice by oral inoculation with Escherichia coli O86:B7 bacteria. Infect Immun 70: 6215–6222. 12379700 10.1128/IAI.70.11.6215-6222.2002 PMC130328

[26] Mehta A, Block TM (2008) Fucosylated glycoproteins as markers of liver disease. Dis Markers 25: 259–265. 19126969 10.1155/2008/264594 PMC3827789

[27] Papp M, Norman GL, Vitalis Z, Tornai I, Altorjay I, et al (2010) Presence of anti-microbial antibodies in liver cirrhosis–a tell-tale sign of compromised immunity? PLoS ONE 5: e12957. 20886039 10.1371/journal.pone.0012957 PMC2944893

[28] Sandler NG, Koh C, Roque A, Eccleston JL, Siegel RB, et al.. (2011) Host response to translocated microbial products predicts outcomes of patients with HBV or HCV infection. Gastroenterology 141: 1220–1230, 1230 e1221–1223. 10.1053/j.gastro.2011.06.063 PMC3186837 21726511

[29] Galili U, Wang L, LaTemple DC, Radic MZ (1999) The natural anti-Gal antibody. Subcell Biochem 32: 79–106. 10391992 10.1007/978-1-4615-4771-6_4

[30] Wormald MR, Rudd PM, Harvey DJ, Chang SC, Scragg IG, et al (1997) Variations in oligosaccharide-protein interactions in immunoglobulin G determine the site-specific glycosylation profiles and modulate the dynamic motion of the Fc oligosaccharides. Biochemistry 36: 1370–1380. 9063885 10.1021/bi9621472

[31] Nimmerjahn F, Anthony RM, Ravetch JV (2007) Agalactosylated IgG antibodies depend on cellular Fc receptors for in vivo activity. Proc Natl Acad Sci U S A 104: 8433–8437. 17485663 10.1073/pnas.0702936104 PMC1895967

[32] Potter BJ, Trueman AM, Jones EA (1973) Serum complement in chronic liver disease. Gut 14: 451–456. 4737028 10.1136/gut.14.6.451 PMC1412744

[33] LaTemple DC, Henion TR, Anaraki F, Galili U (1996) Synthesis of alpha-galactosyl epitopes by recombinant alpha1,3galactosyl transferase for opsonization of human tumor cell vaccines by anti-galactose. Cancer Res 56: 3069–3074. 8674064

[34] Parker W, Lin SS, Yu PB, Sood A, Nakamura YC, et al (1999) Naturally occurring anti-alpha-galactosyl antibodies: relationship to xenoreactive anti-alpha-galactosyl antibodies. Glycobiology 9: 865–873. 10460828 10.1093/glycob/9.9.865

[35] Parker W, Stitzenberg KB, Yu PB, Pratt VS, Nakamura YC, et al (2001) Biophysical characteristics of anti-Gal(alpha)1–3Gal IgM binding to cell surfaces: implications for xenotransplantation. Transplantation 71: 440–446. 11233908 10.1097/00007890-200102150-00018

[36] Yu PB, Holzknecht ZE, Bruno D, Parker W, Platt JL (1996) Modulation of natural IgM binding and complement activation by natural IgG antibodies: a role for IgG anti-Gal alpha1–3Gal antibodies. J Immunol 157: 5163–5168. 8943428

[37] Block TM, Comunale MA, Lowman M, Steel LF, Romano PR, et al (2005) Use of targeted glycoproteomics to identify serum glycoproteins that correlate with liver cancer in woodchucks and humans. Proc Natl Acad Sci U S A 102: 779–784. 15642945 10.1073/pnas.0408928102 PMC545516

[38] Comunale MA, Lowman M, Long RE, Krakover J, Philip R, et al (2006) Proteomic analysis of serum associated fucosylated glycoproteins in the development of primary hepatocellular carcinoma. Journal of Proteome Research 6: 308–315. 10.1021/pr050328x 16457596

[39] Guile GR, Rudd PM, Wing DR, Prime SB, Dwek RA (1996) A rapid high-resolution high-performance liquid chromatographic method for separating glycan mixtures and analyzing oligosaccharide profiles. Anal Biochem 240: 210–226. 8811911 10.1006/abio.1996.0351

[40] Rudd PM, Dwek RA (1997) Rapid, sensitive sequencing of oligosaccharides from glycoproteins. Curr Opin Biotechnol 8: 488–497. 9265730 10.1016/s0958-1669(97)80073-0

[41] Rudd PM, Guile GR, Kuster B, Harvey DJ, Opdenakker G, et al (1997) Oligosaccharide sequencing technology. Nature 388: 205–207. 9217165 10.1038/40677

[42] Rudd PM, Mattu TS, Zitzmann N, Mehta A, Colominas C, et al (1999) Glycoproteins: rapid sequencing technology for N-linked and GPI anchor glycans. Biotechnol 16: 1–21. 10.1080/02648725.1999.10647969 10819075

[43] Rudd PM, Endo T, Colominas C, Groth D, Wheeler SF, et al (1999) Glycosylation differences between the normal and pathogenic prion protein isoforms. Proc Natl Acad Sci U S A 96: 13044–13049. 10557270 10.1073/pnas.96.23.13044 PMC23897

